# Case Report: Pericardial Effusion Treated With Pericardiectomy Plus Right Atrial Mass Resection: A 2-Year Follow-Up of Cardiac Rosai-Dorfman Disease

**DOI:** 10.3389/fcvm.2021.668031

**Published:** 2021-05-31

**Authors:** Edoardo Conte, Antonio Brucato, Francesco Petrella, Emanuela Passoni, Gianfranco Lauri, Mauro Bigliardi, De Camilli Elisa, Gabriella Ricciardi, Carlo Selmi, Piergiuseppe Agostoni, Francesco Alamanni, Daniele Andreini

**Affiliations:** ^1^Centro Cardiologico Monzino, Istituto di Ricerca e Cura a Carattere Scientitico (IRCCS), Milan, Italy; ^2^Department of Biomedical Sciences for Health, University of Milan, Milan, Italy; ^3^Fatebenefratelli Hospital, University of Milan, Milan, Italy; ^4^IRCCS European Institute of Oncology, Milan, Italy; ^5^Department of Oncology and Hemato-Oncology, University of Milan, Milan, Italy; ^6^Operative Unit of Dermatology, IRCCS Foundation Ca' Granda Ospedale Maggiore Policlinico, Milan, Italy; ^7^ASST-Azienda Socio Sanitaria Territoriale di Mantova, Milan, Italy; ^8^Rheumatology, Humanitas Clinical and Research Center – IRCCS, Milan, Italy; ^9^Department of Biomedical Science, Humanitas University of Milan, Milan, Italy; ^10^Cardiovascular Section, Department of Clinical Sciences and Community Health, University of Milan, Milan, Italy

**Keywords:** Rosai-Dorfman disease, pericardial effusion, pericardiectomy, case report, right atrial mass

## Abstract

**Background:** Rosai-Dorfman disease (RDD) is rare a sinus histiocytosis typically causing lymphadenopathy. Heart involvement is anecdotal, and <30 cases of cardiac RDD (cRDD) have been reported so far.

**Case Presentation:** A 46-year old woman with positive clinical history for RDD was admitted to our cardiology department with transthoracic echocardiography diagnosis of severe pericardial effusion and right atrial masses. Pericardiocentesis with catheter insertion was performed 3 days after the admission due to clinical evidence of cardiac tamponade. After 10 weeks of maximal medical therapy for inflammatory pericarditis, including non-steroidal anti-inflammatory drugs (NSAIDs), colchicine, steroids, and anakinra, at least 100 ml of pericardial citric liquid has been daily drained suggesting no clinical improvement. Pericardial liquid analysis demonstrated no malignant cells, but immunohistochemical analysis resulted positive for AE1–AE3, D2–40, S100, and CD68 consistent with an RDD diagnosis. Surgical management was judged clinically indicated, and 2 months after admission, the patient underwent pericardiectomy and debulking of atrial mass with freezing of remaining atrial neoformation. Regular clinical and echocardiography evaluation was performed without pericardial effusion recurrence after 2 years of follow-up.

**Conclusions:** This is the first case ever reported of cRDD who survived after 2 years of follow-up. Pericardiectomy could be feasible and effective for recurrent pericardial effusion in cRDD. Close follow-up and a multidisciplinary environment is needed to take care of cRDD patients.

## Background

Rosai-Dorfman disease (RDD) is a rare clinical condition characterized by histiocyte proliferation causing lymphadenopathy, firstly described in 1969 (1). The cause of the disease is unknown, and diagnosis is based on specific immunohistochemical pattern with histiocytes that result to be S100 positive, CD68 positive, and CD1a negative; these characteristics are always present across all the wide spectrum of disease presentation. Previous reports suggest a potential association with HHV-6 and Epstein-Barr virus without further confirmation. Extra-nodal involvement is very rare, and cardiac involvement (cardiac RDD, cRDD) is anecdotal with <30 cases described so far, mostly in adult. Intra-cardiac masses and pericardial or epicardial involvement have been previously described, but the disease could arise from any cardiac site. Cardiac masses is the most common finding in patients with cRDD (2); among nine previous cases described, seven were treated with complete surgical excision, one with partial surgical excision and medical therapy with steroids, while in one patient, oral steroids was the sole conservative treatment. Of interest, previous reports included only two patients with recurrent pericardial effusion, similar to our patient; both patients died due to cRDD and diagnosis was autoptic. Most of previous reports had follow-up limited to few months after first diagnosis, while the only case treated conservatively showed the absence of mass volume increase after 2 years of follow-up. This case presented with both pericardial effusion and cardiac masses, and pericardiectomy plus cardiac masses partial excision was effectively performed for the first time ever in a patients affected by cRDD.

## Case Report

**Figure 1:**
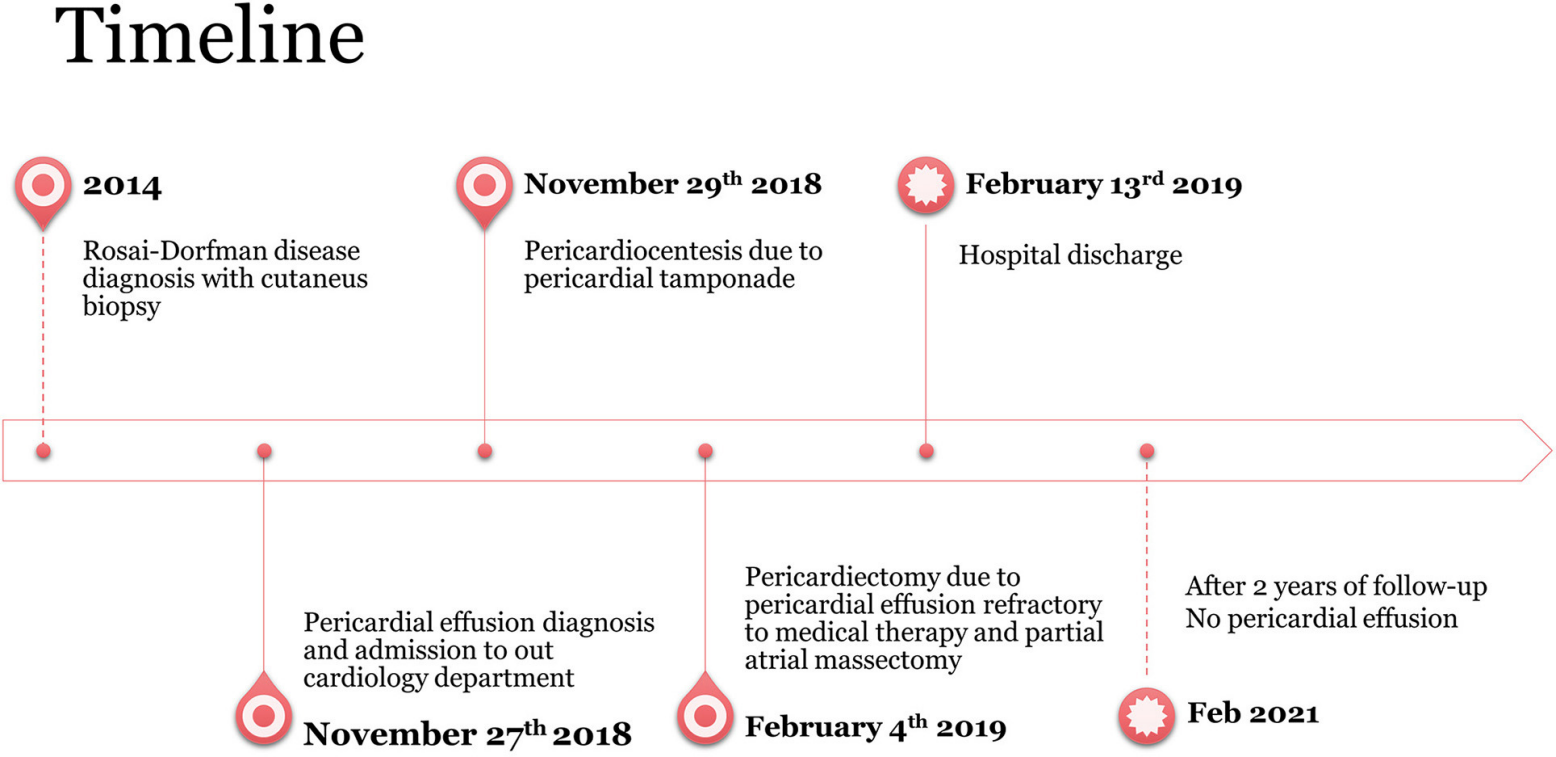
The timeline of the patient's clinical history is presented.

**Figure 2:**
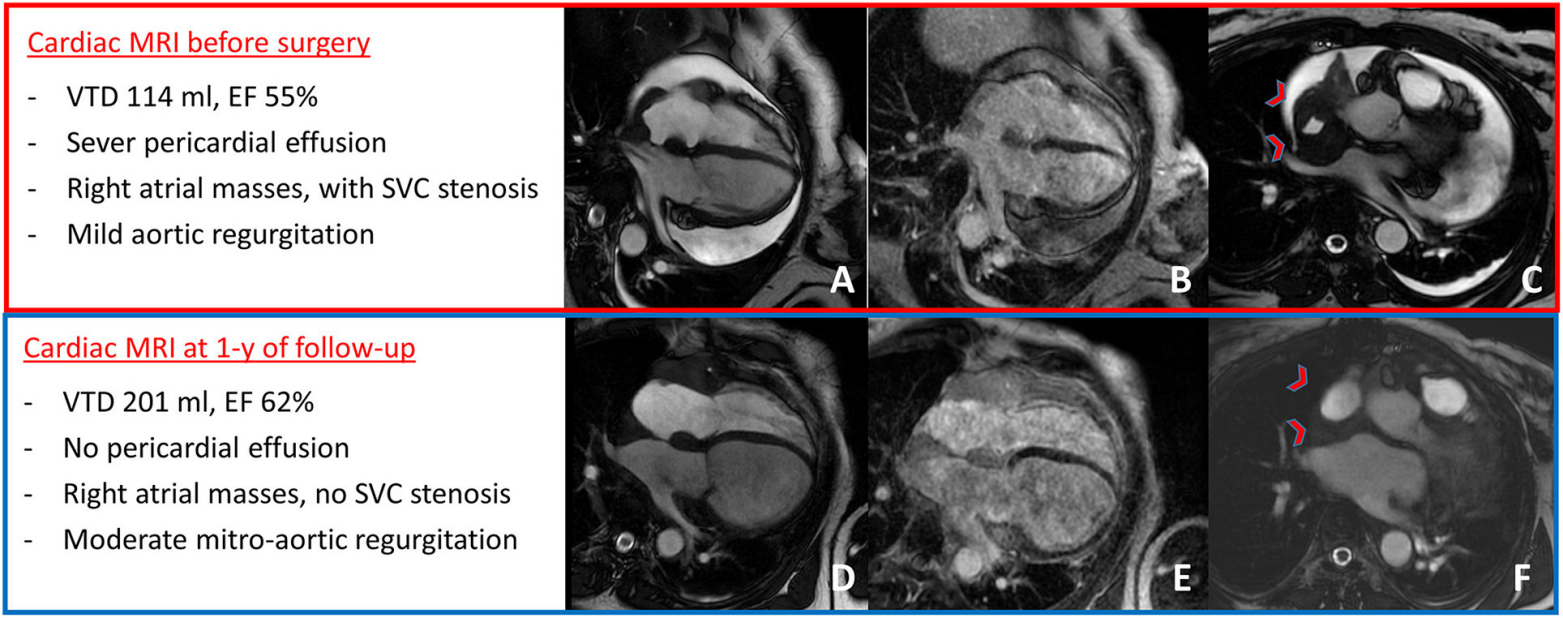
Cardiac MRI at presentation showed severe pericardial effusion with mild aortic regurgitation **(A)**, right atrial masses with LGE **(B)**, and mass infiltration of the superior cava vein causing stenosis [red arrow-heads in **(C)**]. After 1 year of follow-up, cardiac MRI showed no pericardial effusion with moderate mitro-aortic regurgitation **(D)** and mild increase in right atrial masses' size **(E)**, but with no superior cava vein stenosis [red arrow-heads in **(F)**].

A 46-year old woman with positive clinical history for RDD with cutaneous involvement and arthritis, diagnosed 5 years before, was admitted to our cardiology department after transthoracic echocardiography diagnosis of severe pericardial effusion and right atrial masses (Figure 1). At admission, she referred dyspnea during ordinary activity (NYHA III), but no clinical signs of heart failure were present. Cardiac auscultation demonstrated soft heart sounds but pulsus paradoxus was detectable in the presence of sinus tachycardia and normal arterial pressure values (120/70 mmHg). Blood examination showed leukocytosis (WBC 25,000/μl, n.v. <10,000/ul) and increased inflammatory indexes (CRP 47.5 mg/l, n.v. <7 mg/l), while kidney and liver function indexes as well as BNP and Tn-I values were normal. Cardiac MRI confirmed the presence of severe pericardial effusion without concomitant signs of pericardial inflammation. Three separate right atrial neoformations were identified in the interatrial septum and near the right lateral atrioventricular junction (maximum size 13 × 19 mm involving the lateral wall of the right atrium). These masses appeared solid, with smooth borders, but with atrial wall infiltration; of note, superior cava vein involvement with caliber reduction was evident. Masses had inhomogeneous hyperintense signal at T2-weighted images with post-contrast enhancement (both first-pass perfusion images and late gadolinium enhancement). Overall, taking into consideration the positive medical history for RDD, cardiac MRI findings were consistent with cardiac involvement of RDD [(3, 4); Figure 2]. Possible differential diagnosis was primarily represented by cardiac metastasis from a primitive oncological disease, taking into consideration that this is the most common category of cardiac tumor, especially if multiple lesions are documented in the presence of pericardial effusion, like in this case. However, a total body positron emission tomography was performed and resulted negative for active oncological lesion.

Due to clinical evidence of cardiac tamponade 3 days after hospital admission, pericardiocentesis with catheter insertion was performed. Pericardial fluid analysis demonstrated no malignant cells, and immunohistochemical analysis showed the presence of histiocytes positive for AE1–AE3, D2–40, S100, and CD68 consistent with an RDD diagnosis.

After a total of 10 weeks of maximal medical therapy for inflammatory pericarditis including non-steroidal anti-inflammatory drugs (NSAIDs), colchicine, steroids, and anakinra according to current clinical guidelines, at least 100 ml of pericardial fluid has been daily drained suggesting no clinical improvement.

**Figure 3:**
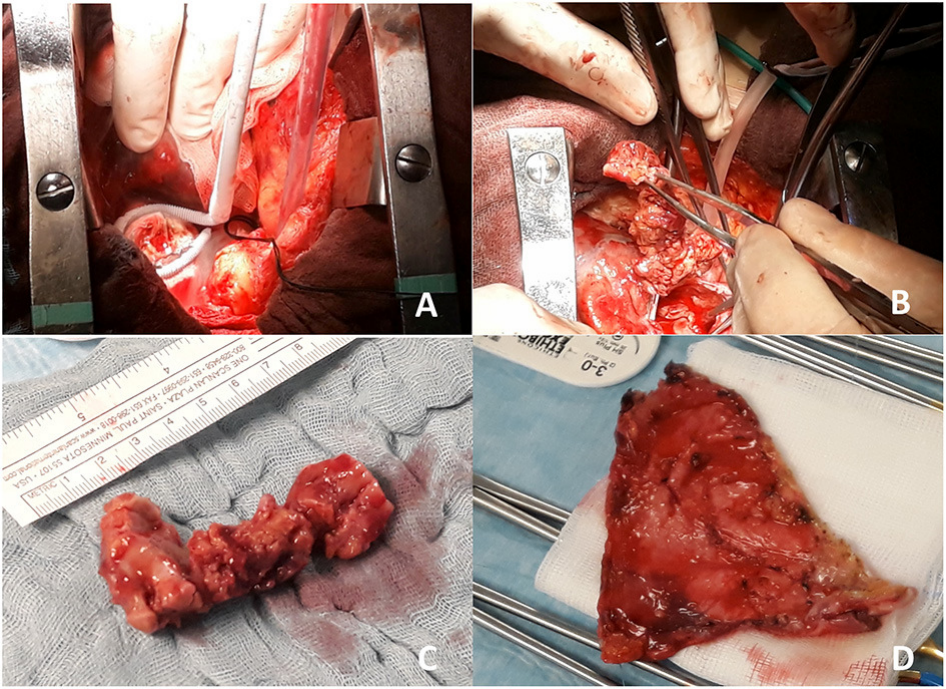
Intraoperative view of residual mass freezing (**A** and Supplementary Video 1) and removal of superior cava vein mass (**B** and Supplementary Video 2). In **(C)**, gross surgical pathology of the removed mass (7 cm length) is shown, while in **(D)**, infiltrated pericardium is shown after pericardiectomy.

**Figure 4:**
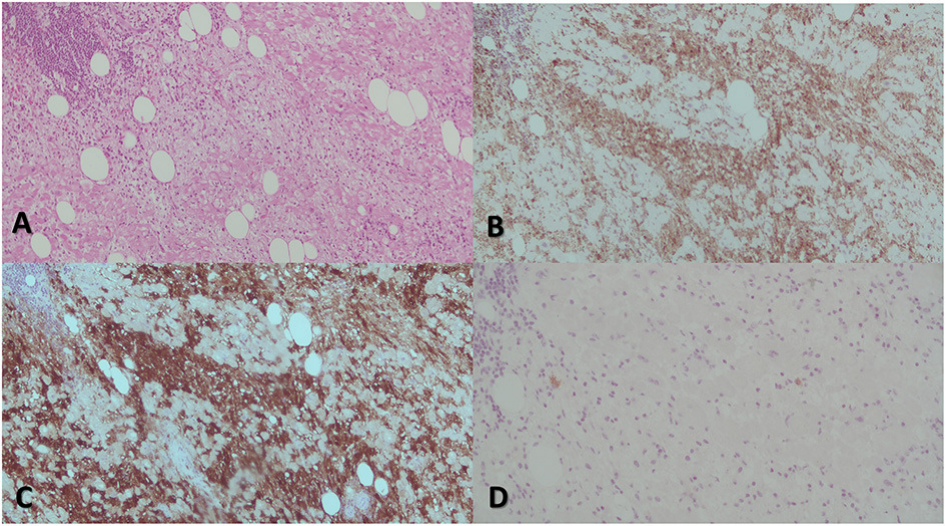
The most prominent histological feature is expansion of the cardiac muscular cells that are filled with histiocytes **(A)**. These characteristically have abundant, often vacuolated cytoplasm, rounded nuclei with coarse chromatin, and a single prominent nucleolus. The atypical histiocytes express CD68 **(B)**. In contrast to reactive sinus histiocytes, they are S100 positive **(C)** but negative for CD1a **(D)**. All these features are consistent with RDD.

**Figure 5:**
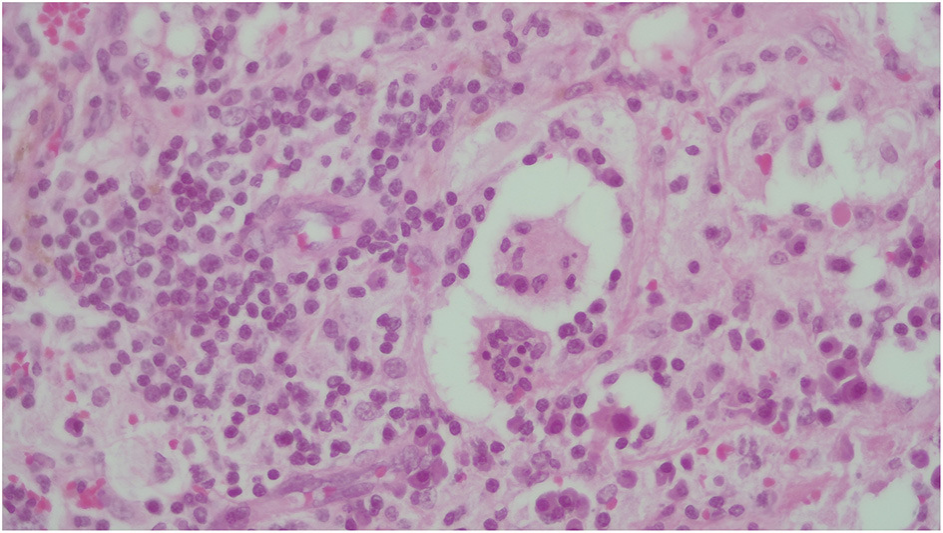
The identification of emperipolesis, defined as the presence of an intact cell within the cytoplasm of another cell. This finding is reported to be typical of RDD and provides a differential diagnosis with PS100+ Erdheim-Chester disease.

Surgical management of recurrent pericardial effusion was judged clinically indicated (5), and 2 months after admission, the patient underwent pericardiectomy and debulking of atrial mass with freezing of remaining atrial neoformation (Figure 3). In this scenario, although radical resection was not achievable, a debulking approach was indicated to prevent mediastinal syndrome due to complete SVC occlusion; this target, in fact, seemed to be more effectively obtained by surgery rather than radiotherapy. After successful resection, the portion of mass involving the superior caval vein was sent for histological analysis and resulted consistent with RDD (Figures 3, 4). More precisely, the characteristic histiocytes were found with abundant and vacuolated cytoplasm, rounded nuclei with coarse chromatin, and a single prominent nucleolus. Emperipolesis was demonstrated as well (Figure 5). The post-operative course was uneventful, and the patient was dismissed 2 weeks after surgery in good clinical condition. Regular clinical and echocardiography evaluation was performed, and patient had no pericardial effusion. At follow-up, cardiac MRI confirmed the absence of pericardial effusion, the mild and slow progression of atrial masses in the presence of moderate-to-severe mitral regurgitation, and mild-to-moderate aortic regurgitation that was not present at the time of surgery and is possibly related to heart valve involvement of cRDD (Figure 2).

## Discussion and Conclusion

Rosai-Dorfman is a rare disease causing massive lymphadenopathy due to histiocyte proliferation. Cardiac involvement is anecdotal with <30 cases described. Heart involvement has been previously described as an intra-cardiac mass, pericardial or epicardial involvement, and pulmonary arterial/aorta infiltration. Previous reports included only six patients with pericardial involvement of which three patients had recurrent pericardial effusion; two of them due to cRDD complications (2), while in the third report of patients presenting with pericardial tamponade, no clear information regarding prognosis is provided (6). Of interest, in a previously described case (7) presenting with both pleural and pericardial recurrent effusion, multiple pleural biopsies resulted negative for pathological findings, highlighting how the disease could be a diagnostic challenge even if suspected. Recently, another case of cRDD pericardial presentation (fibrinous pericarditis) has been described after autoptic evaluation, underlining the dismal prognosis of pericardial involvement from the disease, especially when unrecognized (8).

When cardiac masses are present, a complete excision has been previously described (9), but in our patient, it was not feasible due to the extensive atrial wall infiltration involving the tricuspid annulus as well. The present case is the first in which surgery was indicated for intractable recurrent pericardial effusion effectively treated with pericardiectomy; partial removal of the mass was performed as well, in order to avoid the post-surgical progression of superior caval vein stenosis. After 2 years of follow-up, the longest ever reported for cRDD, no pericardial effusion is detectable, confirming that pericardiectomy could be effective in cRDD, but the rapid progression of both mitral and aortic regurgitation from mild to moderate-to-severe entity, possibly due to RDD involvement, pointed out the aggressive nature of the disease itself and the need for close clinical and echocardiographic follow-up in this kind of patients.

In conclusion, even taking into consideration the limited possibility to extrapolate definite therapeutic strategies from a single case report, the main teaching points of the present case are that pericardiectomy could be feasible and effective in patients with RDD with cardiac involvement presenting with recurrent pericardial effusion; on the contrary, the progression of both mitral and aortic regurgitation pointed out the aggressive nature of the disease itself that needs a multidisciplinary evaluation and strict follow-up. Finally, as previously described, heart masses due to RDD seem to have a slow progression rate.

## Data Availability Statement

The original contributions presented in the study are included in the article/Supplementary Material, further inquiries can be directed to the corresponding author/s.

## Ethics Statement

Ethical review and approval was not required for the study on human participants in accordance with the local legislation and institutional requirements. The patients/participants provided their written informed consent to participate in this study. Written informed consent was obtained from the individual(s) for the publication of any potentially identifiable images or data included in this article.

## Author Contributions

EC and DA designed and wrote the first draft of the manuscript. AB, FP, PA, FA, and CS provided senior supervision and critically revised the design and conception of the manuscript. EP, GL, MB, GR, and DE provided important contribution in data collection, data analysis and interpretation. All authors critically revised the manuscript for important intellectual content, approved the final version of the manuscript and agreed to be accountable for all aspect of the work. All authors have a fundamental role in the clinical management of the case report.

## Conflict of Interest

The authors declare that the research was conducted in the absence of any commercial or financial relationships that could be construed as a potential conflict of interest.

## Abbreviations

RDD: Rosai-Dorfman disease
NSAIDs: non-steroidal anti-inflammatory drugs
WBC: white blood cells
CRP: C-reactive protein
cRDD: cardiac RDD.
